# Magnetic nanoparticle film reconstruction modulated by immersion within DMSA aqueous solution

**DOI:** 10.1038/srep18202

**Published:** 2016-03-24

**Authors:** Qing Xiang, Cimei Borges Teixeira, Li Sun, Paulo Cesar Morais

**Affiliations:** 1 Wuhan Technology and Business University, School of Information Engineering, Wuhan 430065, China; 2 Universidade de Brasília, Instituto de Física, Brasília DF 70910-900, Brazil; 3 University of Houston, Department of Mechanical Engineering, Houston TX 77204, USA; 4 Huazhong University of Science and Technology, School of Automation, Wuhan 430074, China

## Abstract

The process of reconstruction of pre-fabricated films comprising maghemite nanoparticles deposited onto flat glass substrates triggered by immersion into aqueous solutions of meso-2,3-dimercaptosuccinic acid (DMSA) at increasing concentration (0.025, 0.050, and 0.100 mol/L) is herein reported. The evolution of this process was assessed by measuring the time (*t*) dependence of the particle analysis histogram width (*W*) extracted from atomic force microscopy images. Furthermore, a physical picture to model the film reconstruction which provides reconstruction time constants associated to single particles (*τ*_1_) and small agglomerates (*τ*_*n*_), the key units associated to the process, ranging from *τ*_1_ = 2.9 and *τ*_*n*_ = 3.4 hour (0.025 mol/L) to *τ*_1_ = 5.1 and *τ*_*n*_ = 4.6 hour (0.100 mol/L) is proposed. The nanoparticle-based film reconstruction triggered by an exogenous stimulus, the use of the *W* versus *t* data to describe the process and the model picture accounting for the recorded data have not been previously reported.

Thin films comprising nanoparticles (NPs) hosted onto flat substrates or structured templates have attracted increasing interest in the last few years, spanning from the onset of superferromagnetism in low-dimensional magnetic NP systems^1^,^2^,^3^,^4^,^5^,^6^ up to fabrication of high performance biosensors^7^,^8^,^9^,^10^,^11^. In addition to the huge variety of NP-based film fabrication approaches available nowadays changes in particle-particle assembly triggered by exogenous stimuli, e.g. pH of the liquid medium the film is immersed in^12^, may add specific functionality while opening up strategies for film engineering not yet explored. Moreover, the film morphology control between distinct scenarios, such as randomly deposited or self-organized growth, is still an experimental challenge^13^,^14^. Following practical and fundamental issues involved in fabrication or reconstruction of NP-based films the available characterization tools and the usefulness of the parameters they provide is a matter of debate^15^,^16^,^17^. Among the characterization techniques atomic force microscopy (AFM) has been widely employed to investigate the above-mentioned films^15^,^16^,^17^,^18^,^19^,^20^,^21^,^22^. From static point of view AFM has been proved to be a very useful technique to assess morphological aspects of NP-based films though it has been poorly explored as a tool to investigate two-dimensional (2D) NP-based films in regard to its growth, self-assembly and reconstruction as film’s roughness and thickness have been the main focus of reports found in the literature^23^,^24^,^25^,^26^,^27^,^28^,^29^. The open question in this regard is whether or not AFM can be successfully used to investigate the time-dependence of a NP-based film reconstruction triggered for instance by exogenous stimuli and which variable extracted from AFM images would be appropriated to describe this process. Moreover, a comprehensive model picture describing distinct scenarios of film reconstruction is not yet available. In this study we report on the use of AFM to address these issues, i.e. to investigate the time-dependence of the self-reconstruction of a two dimensional maghemite nanoparticle (MNP) film (2D-MNP film) comprising native maghemite (γ-Fe_2_O_3_) NPs deposited onto flat glass substrates and subsequently treated with aqueous solution of meso-2,3-dimercaptosuccinic acid (DMSA) at different concentration (the exogenous stimulus), namely 0.025, 0.050 and 0.100 mol/L to produce Sample-L, Sample-I and Sample-H, respectively. Finally, a model picture for film reconstruction based on the particle analysis histogram width (*W*) extracted from AFM data is herein proposed while a derived equation describing the time-dependence (*t*) of the film reconstruction is successfully used to fit the recorded data. Worth mentioning that thiol-containing molecular-dressed NPs, including DMSA-coating, has been explored for nanoparticle assembly, drug carrier applications, and organ targeting^30^,^31^,^32^. Moreover, it has been reported that while dressing NPs with DMSA intra- or inter-connection among NPs via disulfide bridge (S-S) can be found, depending upon the DMSA surface grafting coefficient to favor the former or the latter connection^33^. Influence of medium’s pH and surface grafting coefficient in determining particle assembly has been also reported in the literature^12^,^15^,^34^,^35^.

## Results

### AFM data

**Figure 1:**
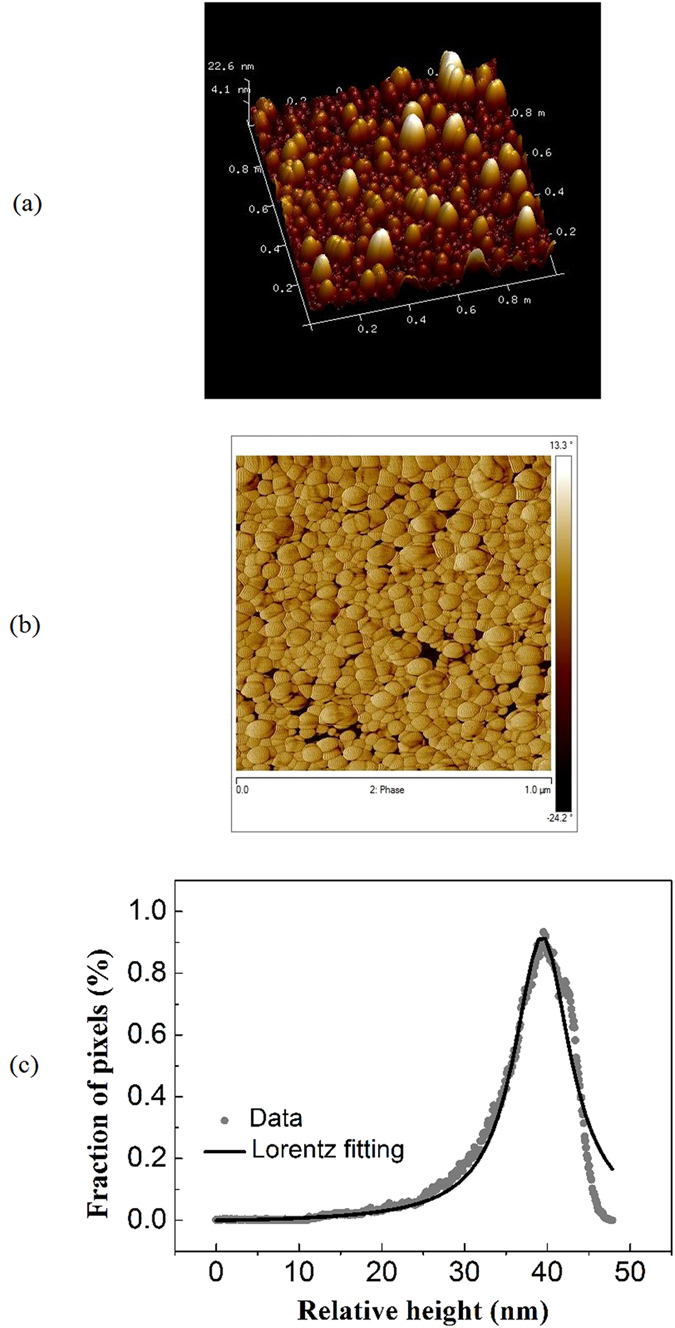


Figure 1a,b show typical 3D and phase AFM images of the DMSA-treated film, respectively. The particle analysis used in the present study provides a method of plotting and analyzing distribution of conjugated pixels identified as particles as a function of relative height within the film^36^. The particle analysis histogram, describing the height distribution of conjugated pixels, can be analyzed using a distribution function thus providing two fitting parameters, namely the center (*h*_*o*_) and the width (*W*) of the distribution, the latter being a measure of surface roughness. We found that the best fit of the particle analysis histograms was achieved using a Lorentzian distribution function, as shown in Fig. 1c for Sample-L (25 hour). Figure 1S (see Supplementary) shows typical 3D and phase AFM images of the DMSA-treated film with the DMSA aqueous solutions (0.025, 0.050, and 0.100 mol/L) at the same cumulative time of 8 hour.

### Particle analysis histogram width versus time

**Figure 2:**
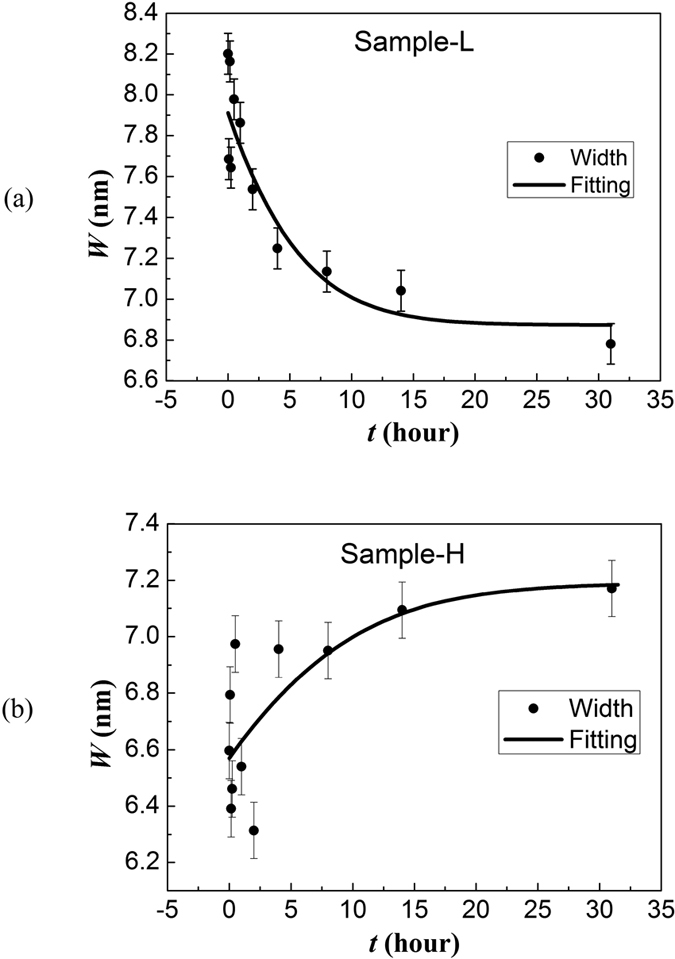
Cumulative time (*t*) dependence of the width parameter (*W*) for the MNP-based films treated with the DMSA solution at: (a) 0.025 mol/L and (b) 0.100 mol/L. Symbols are experimental data whereas the solid lines are fittings using Equation (3).

**Figure 3:**
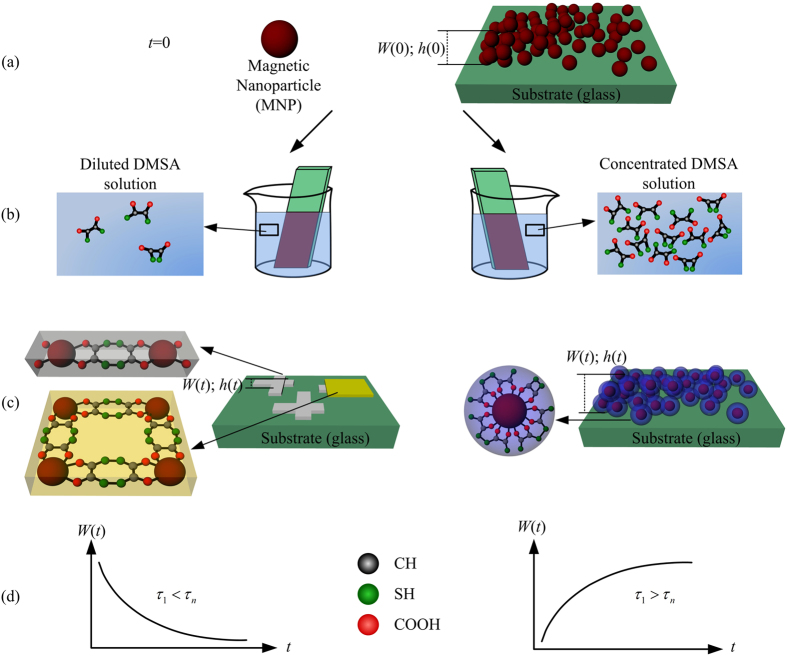
Scheme of main steps: (a) represents the film comprising native nanoparticles, (b) represents the chemical post-treatment of the film, (c) represents the model picture for treatment with diluted DMSA solution leading to inter-particle assembly (left) and with concentrated DMSA solution leading to intra-particle connection (right), (d) represents the general trends of *W* versus *t* data for film reconstruction dominated by DMSA-dressed small agglomerates (left) and single particles (right).

The MNP-based film reconstruction triggered by the DMSA aqueous solution treatment can be assessed by modeling the cumulative time (*t*) dependence of the particle analysis histogram width (*W*). The model picture herein proposed to fit the assessed *W* versus *t* data starts with the argument that while diffusing throughout the 2D-MNP film the DMSA molecule binds onto the native-like maghemite surface via the carboxyl groups^30^,^33^ and triggers the film reconstruction process. There are two limiting scenarios for the proposed model picture we explored in the present report. The first scenario is the film reconstruction based on single (isolated) DMSA-dressed MNPs whereas the second one is the film reconstruction based on agglomerates comprising DMSA-dressed MNPs. In the first scenario the film thickness is expected to increase as a function of time due to the increase of the net diameter of the MNP dressed with the DMSA surface layer, following its return back to the flat substrate and there sticking as randomly as the previous native MNPs. In the second scenario the film thickness decreases as a function of time as the agglomerates are expected to be relatively small (mainly dimers and flat tetramers), forming flat units while settling down and accommodating onto the substrate’s surface. Moreover, small agglomerate formation comprising NPs surface-dressed with thiol-containing moieties has been already reported in the literature^30^. Film treatment with concentrated DMSA aqueous solution leads to high DMSA surface grafting coefficient, bringing S-H groups (DMSA molecule) at the surface of the same nanoparticle close enough to favor the intra-particle oxidation of S-H groups into S-S bridges. In contrast, film treatment using diluted DMSA aqueous solution provides low DMSA surface grafting coefficient, keeping S-H groups located at the surface of the same nanoparticle far apart, not allowing intra-particle oxidation, but enhances inter-particle oxidation among S-H groups of neighboring DMSA-dressed maghemite nanoparticles, thus building small clusters^30^,^33^. Experimental evidences of small agglomerates of magnetic nanoparticles within stable magnetic fluid (MF) samples under zero applied magnetic field is found in the literature^37^,^38^. Small and flat agglomerates consisting of three MNPs in suspension are not expected due to frustration in orienting neighboring magnetic moments within the agglomerate. However, flat tetramers of MNPs can be spontaneously formed in suspension by binding together two dimers with their magnetic moments properly oriented. Further, more likely assisted by magnetic dipolar interaction small agglomerates comprising DMSA-dressed MNPs are easily self-assembled while returning back to the flat substrate. Calculation of magnetic dipolar interaction energy within the flat small agglomerates (3–6 units) comprising magnetic nanoparticles shows that attractive magnetic energy per particle in trimers and hexamers is stronger than in tetramers and pentamers (see Table 1S in Supplementary). The data presented in Fig. 2a,b represent the limiting scenarios, meaning surface reconstruction dominated by small agglomerates comprising DMSA-dressed MNPs and single DMSA-dressed MNPs, respectively. Symbols in Fig. 2a,b describe the cumulative time (*t*) dependence of the particle analysis histogram width (*W*) for Sample-L and Sample-H, respectively. Solid lines in Fig. 2a,b represent the best curve fitting of the experimental data using Equation (3). The fitting values we found for the reconstruction time constant corresponding to the data presented in Fig. 2a,b are: Sample-L (*τ*_1_ = 2.9 hour, *τ*_*n*_ = 3.4 hour) and Sample-H (*τ*_1_ = 5.1 hour, *τ*_*n*_ = 4.6 hour). These values clearly indicate that the film reconstruction is dominated by small agglomerates in Sample-L, as single particles are quickly (*τ*_1_ = 2.9 hour) accommodated within the DMSA-treated film whereas small agglomerates take a longer reconstruction time (*τ*_*n*_ = 3.4 hour). The opposite scenario occurs for Sample-H in which small agglomerates present short reconstruction time (*τ*_*n*_ = 4.6 hour) whereas single particles dominate the surface reconstruction process revealing long reconstruction time (*τ*_1_ = 5.1 hour). Also, it is worth comparing the experimental *W* values extracted from the DMSA-treated film at the shortest (5 minute) and longest (31 hour) times of treatment, namely 6.8 and 7.2 (Sample-H) and 7.7 and 6.9 (Sample-L), respectively. These numbers show a larger relative variation of *W* (about 11%) for Sample-L than for Sample-H (about 6%), signaling a correlated film reconstruction process dominated by agglomerates in Sample-L as opposed to a film reconstruction process in Sample-H dominated by single particles not correlated to one-another while defining the end film morphology. The *W* versus *t* data obtained for Sample-I more likely represent the intermediated scenario in between the two limiting regimes, i.e. it might reflect the balanced competition among single particles and small agglomerates. This sort of balanced competition certainly leads to a complicate mass-transfer process between bulk solution and substrate’s surface which indeed results in large errors associated to the assessed *W* versus *t* data (see Fig. 2S in Supplementary), limiting the possibility of a reasonable fitting. Taken the experimental uncertainty into account the fitting of the *W* versus *t* data collected from Sample-I was not considered. From top to bottom Fig. 3 shows schematically the experimental steps, the model picture representation and the general trend of the experimental data. Figure 3a shows the MNP-based film onto the glass substrate whereas Fig. 3b shows the two extreme scenarios of film reconstruction explored in this report. The left panel in Fig. 3c emphasizes the model picture for treatment with diluted DMSA solution in which inter-particle assembly provides small agglomerates for film reconstruction. Differently, the right panel of Fig. 3c shows the dominant scenario for treatment with concentrated DMSA solution, leading to intra-particle molecular connection which provides single particles for film reconstruction. Left and right panels in Fig. 3d show the general trend of *W* versus *t* data for film reconstruction dominated by small agglomerates and single particles, respectively. As pointed out above, while DMSA-dressed small agglomerates tend to be more densely-packed onto the substrate’s surface, resulting in systematic decreasing of *W* as a function of *t*, film reconstruction based on DMSA-dressed single particles results in systematic increasing of the *W* versus *t* curve.

In summary, two-dimensional films consisting of native maghemite nanoparticles (MNPs) were processed for reconstruction by dipping into aqueous solutions of meso-2,3-dimercaptosuccinic acid (DMSA) at increasing concentration (0.025, 0.050, and 0.100 mol/L). The time evolution of the particle analysis histogram extracted from atomic force microscopy images is pioneering used to provide a physical picture of the MNP-based film reconstruction mechanism. Moreover, the film reconstruction was successfully described by a proposed model which includes single and small agglomerates of DMSA-dressed MNPs. The proposed model was used to assess the typical reconstruction time constants (*τ*_1_ and *τ*_*n*_) by fitting the width (*W*) of the particle analysis histogram as a function of the cumulative DMSA-treatment time (*t*).

## Methods

### Experimental description

The chemical co-precipitation route was used to synthesize the native MNP used in this study, as described in the literature^33^. The maghemite-based film was deposited onto glass slides using a single-step immersion of the flat substrate into ionic magnetic fluid (MF) comprising the as-synthesized MNP (9.5 ± 0.2 nm average core diameter) suspended in low pH aqueous medium, containing about 1 × 10^16^ particle/mL. For film fabrication the stock MF sample was diluted down to 5 × 10^13^ particle/mL whereas the glass substrate was immersed into the MF for 3 minute. Typical AFM image of the fabricated film is shown in Figure 3S (see Supplementary), revealing a high nanoparticle surface coverage, likely above 90% as expected from a first order kinetics film deposition process^39^. Subsequently, after drying out the solvent, the as-prepared MNP-based film was immersed into DMSA aqueous solutions prepared at three different molar concentration, namely 0.025, 0.050 and 0.100 mol/L to produce Sample-L, Sample-I and Sample-H, respectively. The glass substrates containing the as-prepared MNP-based film were immersed into the DMSA solutions at increasing periods of time, up to cumulative time of 30 hour. At the end of each cumulative time the substrates were removed out from the DMSA solution, dried with Nitrogen gas and submitted to AFM imaging.

### Physical model

The model picture introduced to account for the *W* versus *t* data extracted from the AFM images includes three parameters: the DMSA aqueous solution concentration (*M*) used to trigger the 2D-MNP film reconstruction; the cumulative film reconstruction time (*t*) and the contribution of single particles (*S*_1_) and small agglomerates (*S*_*n*_) to the film reconstruction process. The source parameter (*S*) related to single particles (*S*_1_) and small agglomerates (*S*_*n*_), meaning the content of DMSA-dressed single particles and agglomerates available nearby the substrate’s surface while the reconstruction process takes place is introduced. Actually, we claim that the ratio *S*_*n*_/*S*_1_ scales with the DMSA aqueous solution concentration (*M*). This assumption (*S*_*n*_/*S*_1_ ≈ *M*) is based on a recent finding regarding the intra- or inter-particle disulfide bridge formation while the DMSA molecule decorates the MNP’s surface^33^. As described in the literature the intra-particle (inter-particle) disulfide bridge sets in at high (low) DMSA concentration^33^. Therefore, low *M* values favor agglomerates (*S*_*n*_ >> *S*_1_) whereas high *M* values favor single particles (*S*_1_ >> *S*_*n*_). Finally, we argue that the parameter driving the 2D-MNP film reconstruction is the difference in magnetic unit content (*S*_*n*_ or *S*_1_) between the DMSA-treated (*S*_*n*,1_) and the native-like (*S*_*n*’,1’_) units. This means that the rate at which film reconstruction takes place, *df*_*n*_/*dt*, scales with the corresponding source parameter, *S*_1_ and *S*_*n*_. Then, the proposed reconstruction film function, *f*_*n*_, is described by:




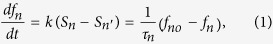




where (*S*_*n*_–*S*_*n’*_) in Equation (1) means the gradient of magnetic units between DMSA-dressed (*S*_*n*_) and DMSA-undressed (*S*_*n’*_) units whereas *τ*_*n*_ is the typical reconstruction time constant. Actually, the reconstruction film function obtained by solving Equation (1), 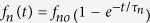 , governs the time-dependence of the end film morphology. From this argument the reconstruction film function can be used to introduce the film’s filling factor, *φ*_*n*_:




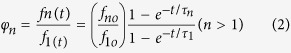




From Equation (2) the higher the DMSA-dressed agglomerate content the bigger the film’s filling factor (the lower the roughness). Then, the broadening of the film thickness (*σ*_*n*_ = 1/*φ*_*n*_) with respect to the perfectly-packed surface morphology linearly scales with the experimentally obtained width (*W*) shown in Fig. 2a,b (vertical axis) and can be described by:




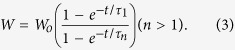




Equation (3) above was used to fit the *W* versus *t* data in order to extract the typical reconstruction time constants (*τ*_1_ and *τ*_*n*_), with *W*_*o*_ equals to 7.2 and 6.9 for Sample-H and Sample-L, respectively.

## Additional Information

**How to cite this article**: Xiang, Q. *et al*. Magnetic nanoparticle film reconstruction modulated by immersion within DMSA aqueous solution. *Sci. Rep.* **6**, 18202; doi: 10.1038/srep18202 (2016).

## Supplementary Material

Supplementary Information
