# Supplementary material for: Magnetic nanoparticle film reconstruction modulated by immersion within DMSA aqueous solution
Source: Sci Rep. 2016 Mar 24;6:18202. doi: 10.1038/srep18202 (PMC4806361; doi:10.1038/srep18202)
Supplement: Supplementary Information [file srep18202-s1.pdf]

Magnetic nanoparticle film reconstruction modulated by immersion within DMSA aqueous solution

Qing Xiang[1], Cimei Borges Teixeira,[2] Li Sun,[3] Paulo Cesar Morais[4,2,*]

[1]Wuhan Technology and Business University, School of Information Engineering, Wuhan 430065, China

[2]Universidade de Brasília, Instituto de Física, Brasília DF 70910-900, Brazil

[3]University of Houston, Department of Mechanical Engineering, Houston TX 77204, USA

[4]Huazhong University of Science and Technology, School of Automation, Wuhan 430074, China

*Corresponding author: Paulo Cesar Morais, E-mail: moraispc@aol.com

Supplementary

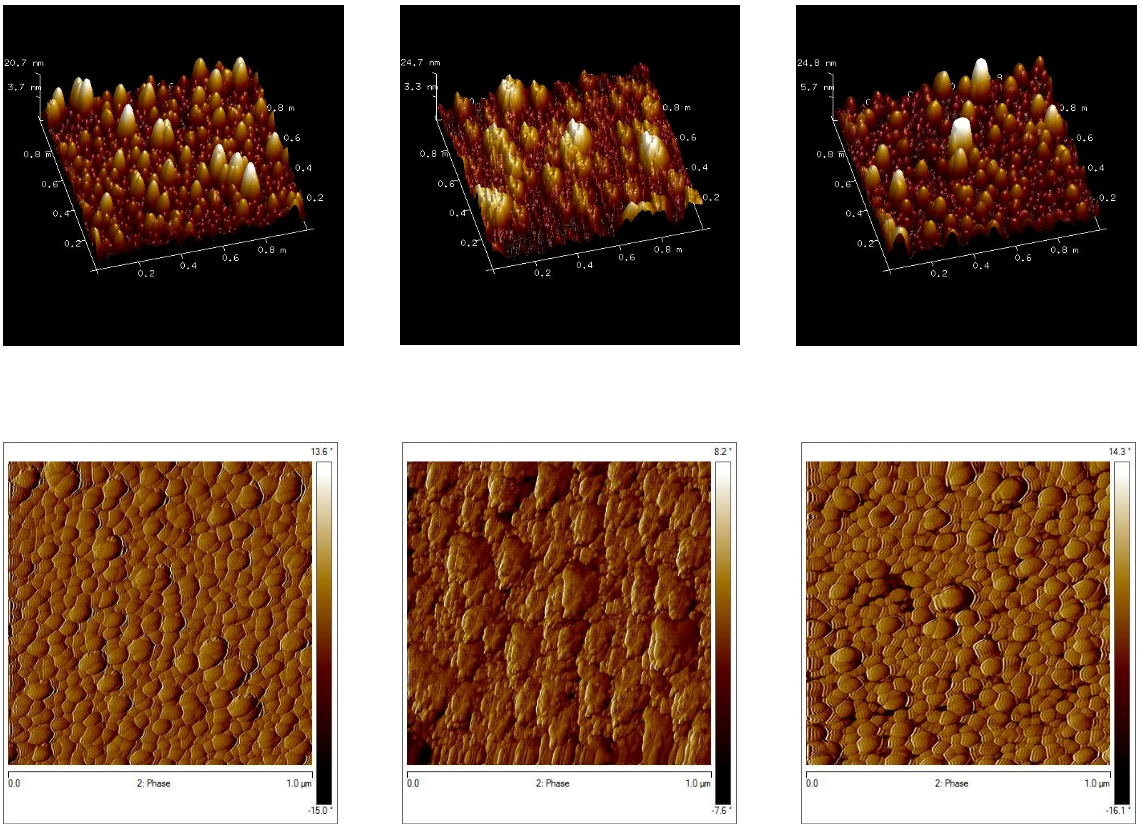

Figure 1S| Typical 3D (top) and phase (bottom) AFM images of the DMSA-treated films using DMSA aqueous solutions at 0.025 mol/L (left panel), 0.050 mol/L (middle panel) and 0.100 mol/L (right panel) at the same cumulative time of 8 hour.

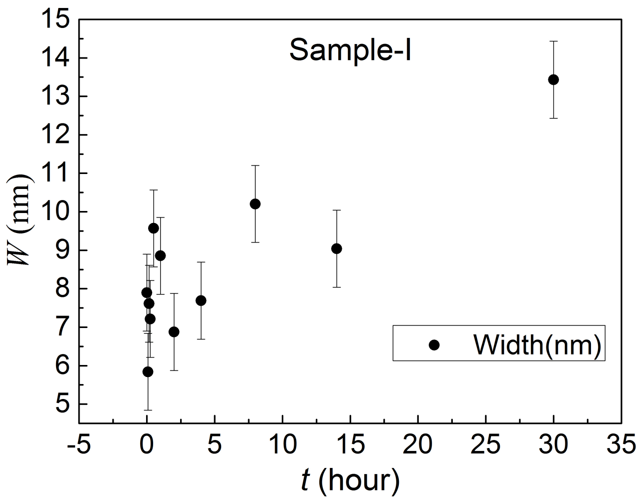

Figure 2S| Cumulative time (*t*) dependence of the width parameter (*W*) for the MNP-based film treated with the DMSA solution at 0.050 mol/L. Symbols represent experimental data.

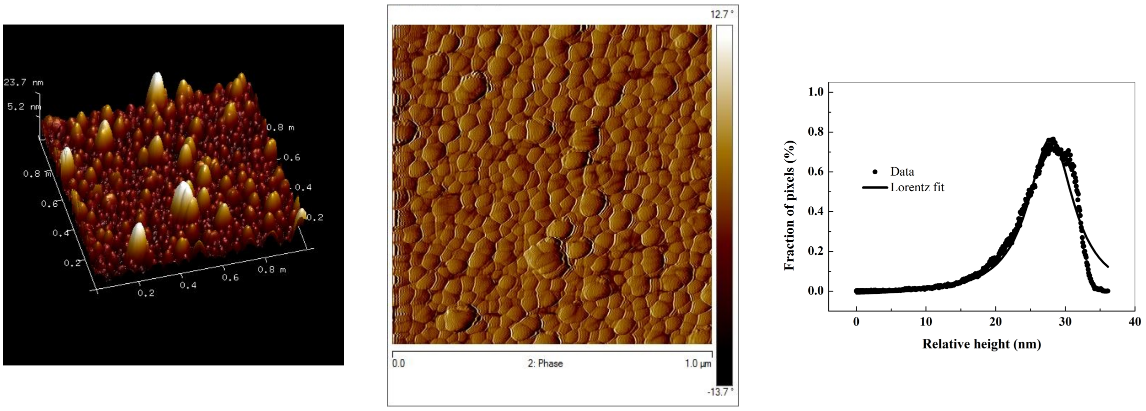

Figure 3S| Typical 3D (left panel) and phase (middle panel) AFM images of the DMSA-untreated film after immersion within the diluted MF sample (5×10^{13} particle/mL) for 3 minute. The right panel shows the corresponding particle analysis histogram data (symbols) and the curve fitting using the Lorentzian distribution function (solid line).

Table 1S| Calculated values of highest magnetic dipole attractive energy (E_d) per particle within dimer and small (3-6) flat agglomerates comprising identical magnetic nanoparticles. Calculation was performed for out-of-plane orientation of magnetic moments (up and down) and is indicated in units of m^2/d^3 (*m*: particle's magnetic moment; *d*: particle's diameter).

Structure	dimer	trimer	tetramer	pentamer	hexamer
Number of particles	2	3	4	5	6
E_d/particle (m^2/d^3)	-0.50	-0.33	-0.68	-0.55	-0.87
